# Supplementary material for: Decomposition of income-related inequality in health check-ups services participation among elderly individuals across the 2008 financial crisis in Taiwan
Source: PLoS One. 2021 Jun 10;16(6):e0252942. doi: 10.1371/journal.pone.0252942 (PMC8192017; doi:10.1371/journal.pone.0252942)
Supplement: S1 Table — (DOCX) [file pone.0252942.s001.docx]

S1 Table. Correlation matrix of independent variables

|  | Premed | lpinco | Sex | Ageg | Edu | Number of individuals living together | Marr | Drink | Smoke | Chew | Exercise | Self-rated health | With Chronic disease | Mobility |
| --- | --- | --- | --- | --- | --- | --- | --- | --- | --- | --- | --- | --- | --- | --- |
| premed | 1 |  |  |  |  |  |  |  |  |  |  |  |  |  |
| lpinco | 0.0890 | 1 |  |  |  |  |  |  |  |  |  |  |  |  |
| Sex | 0.0067 | 0.1526 | 1 |  |  |  |  |  |  |  |  |  |  |  |
| Ageg | 0.0109 | 0.0445 | 0.0421 | 1 |  |  |  |  |  |  |  |  |  |  |
| Edu | 0.0863 | 0.1818 | 0.3790 | -0.0554 | 1 |  |  |  |  |  |  |  |  |  |
| Number of individuals living together | -0.0440 | -0.0615 | -0.0223 | -0.0641 | -0.0729 | 1 |  |  |  |  |  |  |  |  |
| Marr | 0.0594 | 0.0293 | 0.2728 | -0.2357 | 0.1917 | 0.0938 | 1 |  |  |  |  |  |  |  |
| Drink | -0.0102 | 0.0776 | 0.3030 | -0.1164 | 0.1642 | -0.0134 | 0.1190 | 1 |  |  |  |  |  |  |
| Smoke | -0.0832 | 0.0438 | 0.4574 | -0.0346 | 0.0993 | 0.0058 | 0.0900 | 0.2482 | 1 |  |  |  |  |  |
| Chew | -0.0295 | 0.0283 | 0.1857 | -0.0810 | 0.0092 | 0.0059 | 0.0426 | 0.1752 | 0.1945 | 1 |  |  |  |  |
| Exercise | 0.0723 | 0.0474 | 0.0622 | -0.0732 | 0.2010 | -0.0357 | 0.0442 | 0.0525 | -0.0159 | -0.0555 | 1 |  |  |  |
| Self-rated health | 0.0067 | 0.0430 | 0.0814 | -0.0434 | 0.1295 | 0.0234 | 0.0611 | 0.1311 | 0.0161 | -0.0160 | 0.0929 | 1 |  |  |
| With Chronic disease | 0.0765 | 0.0186 | -0.0802 | 0.0306 | 0.0090 | -0.0149 | -0.0409 | -0.0755 | -0.0897 | 0.0017 | 0.0503 | -0.1761 | 1 |  |
| Mobility | -0.0193 | -0.0461 | -0.2217 | 0.2408 | -0.1928 | -0.0463 | -0.1800 | -0.1765 | -0.1216 | -0.0437 | -0.1301 | -0.2923 | 0.1324 | 1 |
