# Supplementary material for: Decomposition of income-related inequality in health check-ups services participation among elderly individuals across the 2008 financial crisis in Taiwan
Source: PLoS One. 2021 Jun 10;16(6):e0252942. doi: 10.1371/journal.pone.0252942 (PMC8192017; doi:10.1371/journal.pone.0252942)
Supplement: S2 Table — (DOCX) [file pone.0252942.s002.docx]

S2 Table. Correlation matrix of independent variables, 2005

|  | Premed | lpinco | Sex | Ageg | Edu | Number of individuals living together | Marr | Drink | Smoke | Chew | Exercise | Self-rated health | With Chronic disease | Mobility |
| --- | --- | --- | --- | --- | --- | --- | --- | --- | --- | --- | --- | --- | --- | --- |
| premed | 1 |  |  |  |  |  |  |  |  |  |  |  |  |  |
| lpinco | 0.0924 | 1 |  |  |  |  |  |  |  |  |  |  |  |  |
| Sex | 0.0175 | 0.1865 | 1 |  |  |  |  |  |  |  |  |  |  |  |
| Ageg | 0.0319 | 0.0460 | 0.0257 | 1 |  |  |  |  |  |  |  |  |  |  |
| Edu | 0.0562 | 0.1800 | 0.3846 | -0.0697 | 1 |  |  |  |  |  |  |  |  |  |
| Number of individuals living together | -0.0479 | -0.0679 | -0.0267 | -0.0247 | -0.0621 | 1 |  |  |  |  |  |  |  |  |
| Marr | 0.0300 | 0.0335 | 0.2743 | -0.2563 | 0.2035 | 0.0439 | 1 |  |  |  |  |  |  |  |
| Drink | -0.0142 | 0.0645 | 0.3090 | -0.0979 | 0.1576 | -0.0255 | 0.1119 | 1 |  |  |  |  |  |  |
| Smoke | -0.0608 | 0.0710 | 0.5285 | -0.0178 | 0.1485 | -0.0085 | 0.1093 | 0.2782 | 1.0000 |  |  |  |  |  |
| Chew | -0.0480 | 0.0130 | 0.0901 | -0.0967 | -0.0317 | 0.0001 | -0.0124 | 0.1199 | 0.1743 | 1 |  |  |  |  |
| Exercise | 0.0598 | 0.0664 | 0.0679 | -0.0422 | 0.2056 | -0.0693 | 0.0259 | 0.0397 | -0.0074 | -0.0485 | 1 |  |  |  |
| Self-rated health | 0.0125 | 0.0487 | 0.0851 | -0.0571 | 0.1301 | 0.0446 | 0.0562 | 0.1360 | 0.0065 | -0.0244 | 0.0806 | 1 |  |  |
| With Chronic disease | 0.0761 | 0.0255 | -0.0699 | 0.0142 | 0.0256 | -0.0301 | -0.0409 | -0.0553 | -0.0758 | -0.0103 | 0.0549 | -0.1755 | 1 |  |
| Mobility | -0.0250 | -0.0592 | -0.2339 | 0.2162 | -0.2160 | -0.0422 | -0.1696 | -0.1621 | -0.1320 | -0.0117 | -0.1028 | -0.3007 | 0.1361 | 1 |
