# Supplementary material for: Decomposition of income-related inequality in health check-ups services participation among elderly individuals across the 2008 financial crisis in Taiwan
Source: PLoS One. 2021 Jun 10;16(6):e0252942. doi: 10.1371/journal.pone.0252942 (PMC8192017; doi:10.1371/journal.pone.0252942)
Supplement: S3 Table — (DOCX) [file pone.0252942.s003.docx]

S3 Table. Correlation matrix of independent variables, 2009

|  | Premed | lpinco | Sex | Ageg | Edu | Number of individuals living together | Marr | Drink | Smoke | Chew | Exercise | Self-rated health | With Chronic disease | Mobility |
| --- | --- | --- | --- | --- | --- | --- | --- | --- | --- | --- | --- | --- | --- | --- |
| premed | 1 |  |  |  |  |  |  |  |  |  |  |  |  |  |
| lpinco | 0.0583 | 1 |  |  |  |  |  |  |  |  |  |  |  |  |
| Sex | 0.0145 | 0.1321 | 1 |  |  |  |  |  |  |  |  |  |  |  |
| Ageg | -0.0169 | 0.0335 | 0.0631 | 1 |  |  |  |  |  |  |  |  |  |  |
| Edu | 0.0959 | 0.1707 | 0.3866 | -0.0489 | 1 |  |  |  |  |  |  |  |  |  |
| Number of individuals living together | -0.0206 | -0.0325 | -0.0281 | -0.1016 | -0.0749 | 1 |  |  |  |  |  |  |  |  |
| Marr | 0.0877 | 0.0270 | 0.2715 | -0.2168 | 0.1821 | 0.1520 | 1 |  |  |  |  |  |  |  |
| Drink | -0.0003 | 0.1025 | 0.2952 | -0.1324 | 0.1760 | -0.0030 | 0.1260 | 1 |  |  |  |  |  |  |
| Smoke | -0.0628 | 0.0553 | 0.3661 | -0.0396 | 0.0705 | -0.0063 | 0.0671 | 0.2110 | 1 |  |  |  |  |  |
| Chew | -0.0465 | 0.0185 | 0.2700 | -0.0834 | 0.0219 | 0.0276 | 0.0813 | 0.2268 | 0.2852 | 1 |  |  |  |  |
| Exercise | 0.0994 | 0.0404 | 0.0498 | -0.0968 | 0.2067 | -0.0070 | 0.0610 | 0.0628 | -0.0517 | -0.0511 | 1 |  |  |  |
| Self-rated health | 0.0045 | 0.0395 | 0.0766 | -0.0298 | 0.1312 | -0.0037 | 0.0659 | 0.1256 | 0.0257 | -0.0092 | 0.1044 | 1 |  |  |
| With Chronic disease | 0.0577 | -0.0101 | -0.0806 | 0.0390 | -0.0203 | 0.0164 | -0.0398 | -0.0925 | -0.0794 | -0.0070 | 0.0559 | -0.1762 | 1 |  |
| Mobility | -0.0069 | -0.0235 | -0.2148 | 0.2669 | -0.1660 | -0.0570 | -0.1907 | -0.1930 | -0.1285 | -0.0601 | -0.1606 | -0.2848 | 0.1348 | 1 |
