# Supplementary material for: Decomposition of income-related inequality in health check-ups services participation among elderly individuals across the 2008 financial crisis in Taiwan
Source: PLoS One. 2021 Jun 10;16(6):e0252942. doi: 10.1371/journal.pone.0252942 (PMC8192017; doi:10.1371/journal.pone.0252942)
Supplement: S4 Table — (DOCX) [file pone.0252942.s004.docx]

S4 Table. Correlation matrix of independent variables, female, 2005

|  | Predmed | lpinco | Ageg | Edu | Number of individuals living together | Marr | Drink | Smoke | Chew | Exercise | Self-rated health | With Chronic disease | Mobility |
| --- | --- | --- | --- | --- | --- | --- | --- | --- | --- | --- | --- | --- | --- |
| Predmed | 1 |  |  |  |  |  |  |  |  |  |  |  |  |
| lpinco | 0.0635 | 1 |  |  |  |  |  |  |  |  |  |  |  |
| Ageg | -0.0342 | 0.0394 | 1 |  |  |  |  |  |  |  |  |  |  |
| Edu | 0.0402 | 0.1121 | -0.1167 | 1 |  |  |  |  |  |  |  |  |  |
| Number of individuals living together | -0.0623 | -0.0230 | 0.0381 | -0.0552 | 1 |  |  |  |  |  |  |  |  |
| Marr | 0.0583 | -0.0420 | -0.3446 | 0.1172 | 0.0257 | 1 |  |  |  |  |  |  |  |
| Drink | -0.0255 | 0.0378 | -0.0713 | 0.0156 | 0.0185 | 0.0198 | 1 |  |  |  |  |  |  |
| Smoke | -0.0359 | 0.0005 | -0.0066 | -0.0450 | 0.0309 | -0.0761 | 0.0666 | 1 |  |  |  |  |  |
| Chew | -0.0247 | -0.0230 | -0.0410 | -0.0465 | -0.0306 | -0.0490 | 0.1966 | 0.1901 | 1 |  |  |  |  |
| Exercise | 0.0203 | 0.0130 | -0.0769 | 0.1632 | -0.0382 | -0.0120 | -0.0132 | 0.0171 | -0.0241 | 1 |  |  |  |
| Self-rated health | -0.0152 | -0.0172 | -0.0641 | 0.0812 | 0.0453 | 0.0360 | 0.1158 | -0.0131 | 0.0067 | 0.0684 | 1 |  |  |
| With Chronic disease | 0.0156 | 0.0442 | 0.0114 | 0.0356 | -0.0182 | -0.0169 | -0.0298 | -0.0305 | 0.0267 | 0.0842 | -0.1811 | 1 |  |
| Mobility | -0.0178 | 0.0224 | 0.2422 | -0.1431 | -0.0482 | -0.1155 | -0.0582 | 0.0177 | 0.0334 | -0.0749 | -0.3056 | 0.1268 | 1 |
