# Supplementary material for: Decomposition of income-related inequality in health check-ups services participation among elderly individuals across the 2008 financial crisis in Taiwan
Source: PLoS One. 2021 Jun 10;16(6):e0252942. doi: 10.1371/journal.pone.0252942 (PMC8192017; doi:10.1371/journal.pone.0252942)
Supplement: S5 Table — (DOCX) [file pone.0252942.s005.docx]

S5 Table. Correlation matrix of independent variables, female, 2009

|  | premed | lpinco | Ageg | Edu | Number of individuals living together | Marr | Drink | Smoke | Chew | Exercise | Self-rated health | With Chronic disease | Mobility |
| --- | --- | --- | --- | --- | --- | --- | --- | --- | --- | --- | --- | --- | --- |
| premed | 1 |  |  |  |  |  |  |  |  |  |  |  |  |
| lpinco | 0.0330 | 1 |  |  |  |  |  |  |  |  |  |  |  |
| Ageg | -0.0998 | -0.0230 | 1 |  |  |  |  |  |  |  |  |  |  |
| Edu | 0.1125 | 0.1339 | -0.1337 | 1 |  |  |  |  |  |  |  |  |  |
| Number of individuals living together | -0.0263 | -0.0328 | -0.0851 | -0.0509 | 1 |  |  |  |  |  |  |  |  |
| Marr | 0.1084 | -0.0266 | -0.2932 | 0.0770 | 0.1687 | 1 |  |  |  |  |  |  |  |
| Drink | 0.0283 | 0.0753 | -0.1030 | 0.1327 | -0.0329 | 0.0581 | 1 |  |  |  |  |  |  |
| Smoke | -0.0221 | 0.0112 | 0.0182 | -0.0616 | -0.0308 | -0.0332 | 0.0694 | 1 |  |  |  |  |  |
| Chew | -0.0267 | 0.0189 | 0.0036 | -0.0506 | -0.0082 | -0.0508 | 0.2171 | 0.2191 | 1 |  |  |  |  |
| Exercise | 0.0953 | 0.0522 | -0.1425 | 0.1941 | 0.0344 | 0.0441 | 0.0806 | -0.0560 | -0.0656 | 1 |  |  |  |
| Self-rated health | 0.0100 | 0.0045 | -0.0423 | 0.1592 | -0.0042 | 0.0465 | 0.1095 | -0.0510 | -0.0503 | 0.1186 | 1 |  |  |
| With Chronic disease | 0.0339 | -0.0173 | 0.0496 | -0.0400 | 0.0490 | -0.0081 | -0.0660 | -0.0133 | 0.0059 | 0.0257 | -0.1958 | 1 |  |
| Mobility | -0.0516 | 0.0109 | 0.2753 | -0.1137 | -0.0411 | -0.1551 | -0.1058 | -0.0208 | 0.0520 | -0.1599 | -0.2761 | 0.1327 | 1 |
