# Supplementary material for: Decomposition of income-related inequality in health check-ups services participation among elderly individuals across the 2008 financial crisis in Taiwan
Source: PLoS One. 2021 Jun 10;16(6):e0252942. doi: 10.1371/journal.pone.0252942 (PMC8192017; doi:10.1371/journal.pone.0252942)
Supplement: S6 Table — (DOCX) [file pone.0252942.s006.docx]

S6 Table. Correlation matrix of independent variables, male, 2005

|  | Premed | lpinco | Ageg | Edu | Number of individuals living together | Marr | Drink | Smoke | Chew | Exercise | Self-rated health | With Chronic disease | Mobility |
| --- | --- | --- | --- | --- | --- | --- | --- | --- | --- | --- | --- | --- | --- |
| Premed | 1 |  |  |  |  |  |  |  |  |  |  |  |  |
| lpinco | 0.1213 | 1 |  |  |  |  |  |  |  |  |  |  |  |
| Ageg | 0.0962 | 0.0450 | 1 |  |  |  |  |  |  |  |  |  |  |
| Edu | 0.0658 | 0.1280 | -0.0577 | 1 |  |  |  |  |  |  |  |  |  |
| Number of individuals living together | -0.0346 | -0.1065 | -0.0807 | -0.0571 | 1 |  |  |  |  |  |  |  |  |
| Marr | -0.0110 | 0.0129 | -0.1900 | 0.1048 | 0.0842 | 1 |  |  |  |  |  |  |  |
| Drink | -0.0189 | -0.0117 | -0.1413 | 0.0614 | -0.0377 | 0.0398 | 1 |  |  |  |  |  |  |
| Smoke | -0.1097 | -0.0530 | -0.0535 | -0.0866 | -0.0002 | -0.0404 | 0.1605 | 1 |  |  |  |  |  |
| Chew | -0.0670 | 0.0095 | -0.1413 | -0.0893 | 0.0214 | -0.0343 | 0.0610 | 0.1459 | 1 |  |  |  |  |
| Exercise | 0.0970 | 0.1029 | -0.0102 | 0.2254 | -0.0949 | 0.0316 | 0.0403 | -0.0834 | -0.0774 | 1 |  |  |  |
| Self-rated health | 0.0338 | 0.0849 | -0.0556 | 0.1265 | 0.0485 | 0.0331 | 0.1195 | -0.0606 | -0.0551 | 0.0819 | 1 |  |  |
| With Chronic disease | 0.1379 | 0.0339 | 0.0208 | 0.0771 | -0.0446 | -0.0297 | -0.0408 | -0.0581 | -0.0237 | 0.0351 | -0.1617 | 1 |  |
| Mobility | -0.0249 | -0.0587 | 0.2154 | -0.1381 | -0.0514 | -0.1110 | -0.1237 | -0.0210 | -0.0047 | -0.1041 | -0.2767 | 0.1204 | 1 |
