# Supplementary material for: Decomposition of income-related inequality in health check-ups services participation among elderly individuals across the 2008 financial crisis in Taiwan
Source: PLoS One. 2021 Jun 10;16(6):e0252942. doi: 10.1371/journal.pone.0252942 (PMC8192017; doi:10.1371/journal.pone.0252942)
Supplement: S7 Table — (DOCX) [file pone.0252942.s007.docx]

S7 Table. Correlation matrix of independent variables, male, 2009

|  | premed | lpinco | Ageg | Edu | Number of individuals living together | Marr | Drink | Smoke | Chew | Exercise | Self-rated health | With Chronic disease | Mobility |
| --- | --- | --- | --- | --- | --- | --- | --- | --- | --- | --- | --- | --- | --- |
| premed | 1 |  |  |  |  |  |  |  |  |  |  |  |  |
| lpinco | 0.0900 | 1 |  |  |  |  |  |  |  |  |  |  |  |
| Ageg | 0.0822 | 0.0905 | 1 |  |  |  |  |  |  |  |  |  |  |
| Edu | 0.0778 | 0.1260 | -0.0066 | 1 |  |  |  |  |  |  |  |  |  |
| Number of individuals living together | -0.0120 | -0.0235 | -0.1200 | -0.0973 | 1 |  |  |  |  |  |  |  |  |
| Marr | 0.0549 | 0.0204 | -0.1731 | 0.1043 | 0.1623 | 1 |  |  |  |  |  |  |  |
| Drink | -0.0304 | 0.0657 | -0.2067 | 0.0234 | 0.0372 | 0.0485 | 1 |  |  |  |  |  |  |
| Smoke | -0.1080 | 0.0078 | -0.1126 | -0.1134 | 0.0202 | -0.0492 | 0.1317 | 1 |  |  |  |  |  |
| Chew | -0.0730 | -0.0432 | -0.1735 | -0.1345 | 0.0683 | 0.0508 | 0.1432 | 0.2076 | 1 |  |  |  |  |
| Exercise | 0.1034 | 0.0088 | -0.0479 | 0.2175 | -0.0604 | 0.0582 | 0.0300 | -0.0985 | -0.0768 | 1 |  |  |  |
| Self-rated health | -0.0044 | 0.0625 | -0.0263 | 0.0474 | 0.0018 | 0.0489 | 0.1114 | 0.0149 | -0.0244 | 0.0800 | 1 |  |  |
| With Chronic disease | 0.0905 | 0.0251 | 0.0379 | 0.0839 | -0.0324 | -0.0354 | -0.0807 | -0.0800 | 0.0223 | 0.1039 | -0.1424 | 1 |  |
| Mobility | 0.0579 | -0.0034 | 0.3036 | -0.0611 | -0.0971 | -0.1196 | -0.1734 | -0.0803 | -0.0329 | -0.1455 | -0.2757 | 0.1054 | 1 |
