# Supplementary material for: Decomposition of income-related inequality in health check-ups services participation among elderly individuals across the 2008 financial crisis in Taiwan
Source: PLoS One. 2021 Jun 10;16(6):e0252942. doi: 10.1371/journal.pone.0252942 (PMC8192017; doi:10.1371/journal.pone.0252942)
Supplement: Attachment — Submitted filename: Response to Reviewers.docx [file pone.0252942.s009.docx]

Department of Long Term Care, National Quemoy University

No. 1, University Rd., Jinning Township, Kinmen County 89250, Taiwan (R.O.C.)

Point to Point Response letter for “**Decomposition of Income-Related Inequality in Health Check-ups Services Participation Among Elderly Individuals Across the 2008 Financial Crisis in Taiwan**” manuscript

**(PONE-D-20-31185)**

May-06-2021

PLOS ONE

**Journal Requirements:**

**1. Please review your reference list to ensure that it is complete and correct.**

**Response:**

We have checked reference list of this manuscript. We confirmed the reference list is complete and correct.

**2. If you have cited papers that have been retracted, please include the rationale for doing so in the manuscript text, or remove these references and replace them with relevant current references.**

**Response:**

We did not cite papers that have been retracted.

**3. Any changes to the reference list should be mentioned in the rebuttal letter that accompanies your revised manuscript.**

**Response:**

We did not change the reference list.

We have edited the hyperlinks of the doi and PMID of each reference of this manuscript for meeting the publication criteria of PLOS one.

**4. If you need to cite a retracted article, indicate the article’s retracted status in the References list and also include a citation and full reference for the retraction notice.**

**Response:**

We did not cite any retracted articles.

**5. [Note: HTML markup is below. Please do not edit.]
[NOTE: If reviewer comments were submitted as an attachment file, they will be attached to this email and accessible via the submission site. Please log into your account, locate the manuscript record, and check for the action link "View Attachments". If this link does not appear, there are no attachment files.]**

**Response:**

We did not find any reviewers’ comment and did not find attachment file. (Email received on April 30, 2021.)

**6. While revising your submission, please upload your figure files to the Preflight Analysis and Conversion Engine (PACE) digital diagnostic tool,** [**https://pacev2.apexcovantage.com/**](https://pacev2.apexcovantage.com/)**.**

**Response:**

This study is not with any figures. We do not upload figure files.

We did our best to revise the reference list and hope that this revision can meet the publication requirements of PLOS one.

Thank you for your help and patience.

**The following responses to the review comments are based on the review comments received on February 18, 2021.**

**Editor comments:**

We are very grateful to your helpful opinions. Our response for your opinions is as follows:

**1. Please ensure that your manuscript meets PLOS ONE's style requirements, including those for file naming. The PLOS ONE style templates can be found at https://journals.plos.org/plosone/s/file?id=wjVg/PLOSOne_formatting_sample_main_body.pdf and https://journals.plos.org/plosone/s/file?id=ba62/PLOSOne_formatting_sample_title_authors_affiliations.pdf**

**Response:**

We have checked the templates and made the few adjustment to meet the journal requirements.

**2. Please specify in the methods section how demographic information was extracted and stratified for analysis.**

**Response:**

We have specified the data file used in this study, and the variables used in this analysis.

(database part of method section, the second paragraph in page 8 and statistical analysis part of method section, the second paragraph in page 12)

Original

**Database part**

Given the purpose of this study, we analyzed samples of survey respondents aged ≥65 years and estimated the income-related inequality among older adults in health check-up services use.

**Statistical analysis part**

Data analysis employed a probit model. The factors that influence health or preventive care utilization differ between men and women [33-36]; therefore, men and women were separately analyzed to identify potential gender-specific patterns.

Revised

**Database part**

Given the purpose of this study, we analyzed samples of survey respondents aged ≥65 years and selected the personal characteristics, health conditions, health behaviors, preventive care utilization and socioeconomic factors as this study analysis variables.

**Statistical analysis part**

Data analysis employed a probit model. The factors that influence health or health/preventive care utilization differ between men and women [33-38]. We divided respondents into two groups: men and women, and these two groups were separately analyzed in 2005 and 2009.

**3. We note that you have indicated that data from this study are available upon request. PLOS only allows data to be available upon request if there are legal or ethical restrictions on sharing data publicly.**

**Response:**

We confirmed the Implementation Direction on Application and Using NHIS data with National Health Research Institute. NHIS data are unsuitable for public deposition. The implementation direction, conducted by National Health Research Institute, prohibits applicants from publicly depositing NHIS data files in any ways (in the second point of the eighth article). The website of Implementation Directions on Application and Using National Health Interview Survey (NHIS) data files is at <http://nhis.nhri.org.tw/point.html> (in traditional Chinese).

We revised the data available statement as follows:

Original:

The data that support the findings of this study are available from the National Health Research Institutes (NHRI), Taiwan (website: <http://nhis.nhri.org.tw/2009nhis.html>; <http://nhis.nhri.org.tw/2005nhis.html>). Restrictions apply to the availability of these data, which were used under license for the current study, and so are not publicly available. However, data can be available from the corresponding author upon reasonable request.

Revised:

The data that support the findings of this study are available from the National Health Research Institutes (NHRI), Taiwan (website: <http://nhis.nhri.org.tw/2009nhis.html>; <http://nhis.nhri.org.tw/2005nhis.html> ). However, restrictions apply to the availability of these data, which were used under license for the current study, and so are not publicly available. Requests for data can be sent as a formal proposal to the NHRI (<http://nhis.nhri.org.tw>) or contact the person in charge of NHIS data set at [nhisis@nhri.edu.tw](mailto:nhisis@nhri.edu.tw).

**Additional information**

As review’s comment (review 2, No.5 comment), we added the “number of individual living together” variable into the probit analysis model.

The analysis sample size is with a little bit change.

After adding the “number of individual living together” variable in the analysis model,

2005, female group: sample size is from 1229 to 1227.

2009, female group: sample size is from 1417 to 1416.

The sample size is consistent among men group in 2005 and 2009.

**Reviewer comments:**

**Reviewer 1:**

**#1. The authors have addressed an important issue in this manuscript that can influence decision making and policy making in their country.**

**Response:**

Thank for your appreciation and kind opinions.

**#2. This manuscript needs minor language revision. Simplification of the keywords may improve the internet search for other researchers.**

**Response:**

We simplified the key word as following:

“Senior health check-up services” to “health check-up”

“health care inequality” to “inequality”

**#3. Abstract:**

**- The description of the results in the abstract is vague to some extent, better description is already found in the results section of the body of the manuscript.**

**Response:**

Thank you for raising this point. We have revised abstract.

(Abstract, line 13-14, page 3)

Original

The more pro-rich income-related inequality impact of the financial crises on health checkups utilization of the elderly men cannot be ignored, even policy with free health checkups provided by National Health Insurance for seniors in Taiwan.

Revised

The 2008 global financial crisis strengthened the effect on health check-ups use of income-related inequality of elderly men, especially in older adults with negative health behaviors.

Thank you very much for your positive feedback and helpful comments for this manuscript.

**Reviewer comments:**

**Reviewer 2:**

**1. The manuscript states that a “multistage systematic stratified sampling design…” was used. A few lines clarifying precisely what that means with respect to the data used will indeed be helpful (i.e., give the number of sampling stages and details of the various sampling stages). Also, I assumed that the 2005 data and 2009 data are responses from the same set of individuals, but the presentation of the results and discussion indicates I may be wrong. Perhaps the authors may want to clarify this from the onset.**

**Response:**

Thank you for raising this point.

We added more information of sampling design of NHIS (page 7 to 8 in the database part of Materials and Methods section).

The study design is cross-sectional in each year.

We used two cross-sectional datasets (2005 and 2009) as our analysis data.

We revised the description of data set.

(the first line in page 7 in the database part of Materials and Methods section)

Original

Using a cross-sectional study design, we analyzed two datasets, one from 2005 and one from 2009, comprising responses to the Taiwan National Health Interview Survey (NHIS), issued by the Taiwan Health Promotion Administration of the Ministry of Health and Welfare. …………………………………………………….. A multistage systematic stratified sampling design with probability proportional to size sampling was employed, so that the probabilities of the sample were proportional those of the actual population. Given the purpose of this study, we analyzed samples of survey respondents aged ≥65 years and estimated the income-related inequality among older adults in health check-up services use.

Revised

Using a cross-sectional study design, we analyzed two cross-sectional survey datasets, one from 2005 and one from 2009, comprising responses to the Taiwan National Health Interview Survey (NHIS),……………A multistage systematic stratified sampling design with probability proportional to size sampling (PPS) was employed. According to the geographical location, population density and degree of urbanization of the townships/districts, the first step of sampling scheme was to divide 358 townships/districts of Taiwan into 53 strata in 2005 and 48 strata in 2009. The selection probability of township/district was with PPS. For each selected township/district, lins (the smallest administrative unit) were selected with PPS. In each selected lin, respondents were selected with PPS. The total sampling rate is 1.34 ‰ in 2005 and 1.32 ‰ in 2009. Different questionnaire items were used according the age of participants. The completed questionnaires divided into three different data file: under 12 years old, 12-64 years old, and 65 and above years old.

**2. In the CI calculation, a variable called "health status" is defined which the reader is led to believe as meaning "health checkup services utilisation". However, further down in the methods, another variable called "health status" is defined in the section called "Explanatory variables" which I suspect may be referring to something entirely different. Further clarification on this may be needed.**

**Response:**

Thank you for your reminder. We apologize this confusion.

We changed “health status” to “personal health conditions” in the “explanatory variables of definition of variables” part of Materials and Methods section (in page 11).

Original

**Explanatory variables**

Health checkups is a demand for health and lead to further demand for preventive or medical care when necessary [27-29]. Use of health check-up services depends on user’s characters and various other factors [27], that includes demographic, socioeconomic, health status,…………

Revised

**Explanatory variables**

Health checkups is a demand for health and lead to further demand for preventive or medical care when necessary [27-29]. Use of health check-up services depends on user’s characters and various other factors [27], that includes demographic, socioeconomic, personal health conditions,………..

**3. The overall study attempts to pinpoint gender-specific patterns to health checkup services utilisation and, therefore, analyse men and women separately. However, the evidence on which this initial reasoning is based are studies conducted in other populations: Socias et al. 2016, Cameron et al. 2010, Vaidya et al. 2012 and Brunner-Ziegler et al. 2013, are based on Canadian, American and Austrian populations. The same effects may or may not apply to the Taiwan population, and one can only be sure if that is tested empirically. An initial analysis that puts males and females together could be performed to establish whether there is a significant gender difference in health checkup services utilisation. If the authors did this, then it should be acknowledged in the manuscript.**

**Response:**

Thank you for this comment.

We added the literature that analyzes Asian gender separately, such as China, Japan, and South Korea, to get closer to the cultural characteristics of Taiwan's population.

We think, this is a different concept between taking gender as a variable to enter the analysis and separating men and women into two groups to analysis. The former refers that sex as a factor influences the dependent variable (the preventive health utilization in this study), and the latter means that the two groups of men and women have different influencing factors in the use of preventive health utilization.

The analysis at the beginning of this study is to separate men and women group for analysis.

**4. Since some of the health behaviour variables, both positive and negative, may correlate with education level, which could be a source of confounding in the model thus rending some of these variables insignificant. Perhaps the manuscript should acknowledge this and provide data on the correlation between these variables.**

**Response:**

We have provided a correlation matrix between individual variables. It can be found that the correlation between education level and healthy behavior is not high (see supplementary table). The aim of conducting multiple probit regression is to get the partial correlation between the independent variable and the dependent variable, after controlling other confounding variables. And a collinearity of analysis model maybe a considered problem by reviewer. In order to understand whether or not there is a collinearity problem, we also performed a regression diagnosis. The VIF value of the four probit regressions diagnosis results is less than 10. The range of VIF average value is between 1.19 and 1.38 in four analysis models. This VIF value can help us determine that the collinearity problem of regression model can be ignored.

*The results of collinearity diagnosis.*

*2005, female*

*2009, female*

*2005, male*

*2009, male*

Supplement tables (correlation tables)

**5. If data restrictions do not preclude it, I would suggest additional data on respondents’ household (number of individuals in the household, and also a measure of the support available to them at home) should be considered in the model.**

**Response:**

We have added “number of individuals living together” variable into the model analysis, and renew tables of this study. Because this variable is with some missing observations, so the overall sample size in our study is slightly changed.

The effect of this variable (number of individuals living together) is non-statistically significant on the preventive care utilization. The statistical significance of other variables is not changed after introducing “number of individuals living together” variable.

After adding the “number of individual living together” variable into the probit analysis model, the analysis sample size is with a little bit change.

2005, female group: sample size is from 1229 to 1227.

2009, female group: sample size is from 1417 to 1416.

The sample size is consistent among men group in 2005 and 2009.

**6. Tables 3 and 4 are a bit hard to follow. I would suggest they are broken up into two tables each, with one table looking at demography and socioeconomic factors, and the other looking at behaviour and health.**

**Response:**

If table 3 and 4 are disassembled, the number and title of the table will be more complicated.

We classify the variables in Table1, Table 3 and Table 4, and provide the group name of the classification, hoping to provide a better reading experience.

Original

Revised

**7. The effect of income on health checkup services utilisation between males and females is an important result that warrants more discussion than provided in the manuscript. The authors should also provide references to support the statements that "men bear more of a family's financial burden", and "older men have more assets than older women in Taiwan".**

**Response:**

Thank you for this comment. We revised the original inaccurate sentence and added citations to support our argument.

And we delete the "men bear more of a family's financial burden" statement due to no supporting references.

Original:

The reasons may be that men have to bear a more of the family financial burden or older men have more assets than older women in Taiwan

Revised:

The reason may be that the percentage of older men who own assets (54.3%) is higher than that of older women (35.6%) in 2005 in Taiwan [40,41], and the 2007-2008 financial crisis has a direct impact on people with assets [42].

**8. The paper referenced (Cropper, 1997) in discussing the age effect of female health checkup utilisation does not provide sufficient support for the results observed in this manuscript. Cropper reasons that how investment in health (checkups, dietary supplements, etc.) changes over the course of life cannot be determined because of the uncertainty of death. Unlike human capital investment which is high when people are young and declines over time. Moreover, the opposite results obtained for men in both 2005 and 2009 prove the point of Cropper that changes in health investment cannot be determined with any certainty without first assuming the certainty of death. Therefore, I would suggest that the authors discuss those results further and provide relevant support from the literature.**

**Response:**

Thank you for raising this point. We discussed our results with the concept of health-risk effect and lifecycle effect by adding three references: Grossman 1972, Chen 2013, and Hsieh 1997.

(Discussion section, the second paragraph, page 27)

Original

In terms of the age effect on health check-ups utilization, the female participation rate in 2009 is similar to the results of other studies [28], that is, younger groups have a higher health checkups utilization rate than older groups. That is because that the pay-off period of human capital investment among younger group is longer than among older group as Cropper mentioned [39]. However, the result of age effect is the opposite among men group in 2005, that is, older group has a higher health checkups utilization. The possible explanation is that older male group has a gender advantage in our culture, the opinion of pay-off period for human capital investment is not shown among male group. We suggest that future research can further focus on the relationship between gender advantages and the use of preventive health services.

Revised

In terms of the age effect on health check-ups utilization, the opposite results are showed among female and male groups. The female participation rate in 2009 is similar to the results of other studies [28], that is, the 65-74 age group has a higher health checkups utilization rate than group aged 75 and over. This result indicates that the lifecycle effect may dominate the health-risk effect among female group after 2008 financial crisis. The lifecycle effect [43] and the health-risk effect [28, 44] mean that the use of preventive care decrease and increase with age, respectively. However, the result of age effect of among men group is that group aged 75 and over has a higher health checkups utilization than 65-74 age group in 2005, also is similar to some studies in Taiwan [45, 46]. And the age effect of men group shows that the health-risk effect may dominate the lifecycle effect before the 2008 financial crisis. The possible explanation is that older male group has a gender advantage in our culture, the opinion of health-risk effect is not shown among female group. We suggest that future research can further focus on the lifecycle effect/health-risk effect on the use of preventive health services from gender difference perspective.

**9. Lee et al. (2017) did observe similarly that healthy lifestyles lead to an increase in the utilisation of preventive health services. However, there is a clear age difference between the data used in that study and this one. Perhaps acknowledge this?**

**Response:**

We replace this reference with another reference (Cheah, 2018). The age of the research sample of the new alternative literature matches the age of the sample of this study.

**Minor Suggestions**

**m-1. Some of the sentences in the manuscript can benefit from the proper use of commas.**

**Response:**

We use commas and simple sentences as much as possible to make sentences more easier to read.

**m-2. The frequent use of semi-colons in the manuscript makes some sentences unclear or hard to follow. It will help the reader if the semi-colons were to be removed, and such sentences are broken up into two or more simpler sentences.**

**Response:**

We have removed the semi-colons. And broking sentences up into simple sentences.

**m-3. The exact p values should be quoted in the manuscript, instead of just stating “significant” or “marginally significant”.**

**Response:**

We have used p value instead of “significant” or “marginally significant” in page 17.

Original

The income CIs for the use of health check-up services are presented in Table 2. Those with relatively high incomes used health check-up services more than other respondents; this result was statistically significant for both 2005 and 2009. For women, income-related inequality went from concentrated among those with relatively high incomes (marginally significant) in 2005 to less pro-rich concentrated with significantly in 2009; however, a statistically significant change was not evident between study periods.

Revised

Those with relatively high incomes used health check-up services more than other respondents. This result was statistically significant for both 2005 and 2009. For women, income-related inequality went from concentrated among those with relatively high incomes (p value=0.0682) in 2005 to less pro-rich concentrated with significantly (p value=0.0457) in 2009. However, a statistically significant change was not evident between study periods.

**m-4. In some parts of the results and discussion, the manuscript describes people aged 65 – 74 as younger adults and those 75 and over as older adults. I think this description is quite problematic because, for some readers, older adults mean people aged 65 and over. To avoid such confusion, I think the manuscript should quote the exact age group being referred to.**

**Response:**

We have edited this. Thank you for your careful comment.

**m-5. I think. Abstract and page 12 “The CI of older women increased from .0738 in 2005 to .0658 in 2009…” should this be “decreased”?**

**Response:**

Thank you for this comment.

We have corrected it.

**m-6. I think. The last but one sentence of the abstract “…elderly men with negative health behaviours tended to contribution more income-related inequality…” should this be “contribute”?**

**Response:**

We have corrected it.

**m-7. Pages 13 and 14. “The percentage contribution calculating from dividing the absolute contribution…” should this be “calculated”?**

**Response:**

We have corrected it.

Thank you for your careful and helpful comments.
